# Study on the stability and accuracy of the new Booster portable cardiopulmonary function meter

**DOI:** 10.3389/fphys.2024.1453942

**Published:** 2025-01-08

**Authors:** Hezhang Yun, Wenbo Zhang, Chen Yu, Qiang Li, Yafeng Song

**Affiliations:** ^1^ China Institute of Sport and Health Science, Beijing Sport University, Beijing, China; ^2^ School of Physical Education, Zhejiang Guangsha Vocational and Technical University of Construction, Dongyang, China; ^3^ School of Physical Education, Qinghai Normal University, Xining, Qinghai, China

**Keywords:** portable cardiopulmonary function meter, reliability, validity, oxygen uptake, cardiopulmonary function, ventilation volume, wearable devices

## Abstract

This study aims to assess the reliability and accuracy of a novel portable cardiopulmonary function meter, “Booster,” developed by our research group, across various exercise intensities and modalities. The study was segmented into reliability and validity assessments. Twenty-two male participants underwent reliability testing, conducting two sequential tests on a treadmill while wearing the Booster to measure VO_2_ and VE among other parameters at increasing intensities. For validity testing, 64 participants were randomly divided into treadmill and cycle ergometer groups, with tests conducted using both the Booster and the Cortex Metalyzer 3B systems. Overall, the Booster demonstrated high retest reliability for VO_2_ and VE measurements during treadmill exercises, albeit showing poor consistency during rest and low-intensity exercise phases. Validity testing indicated no significant differences in VO_2_ and VE measurements between Booster and Cortex Metalyzer 3B across all exercise stages on both treadmill and cycle ergometer, suggesting good correlation. However, discrepancies in measurements between Booster and Cortex Metalyzer 3B were observed during rest and maximal exertion phases. The Booster exhibits commendable reliability and stability during most treadmill exercise phases and shows generally acceptable validity compared to the Cortex Metalyzer 3B system. Nonetheless, potential measurement discrepancies may occur during rest and maximal exertion conditions.

## 1 Introduction

Assessment of cardiopulmonary function plays a crucial role in exercise physiology, clinical medicine, and health management. Among them, maximal oxygen uptake (VO_2max_) is a vital indicator for evaluating individual cardiopulmonary health (Alexander et al., 2003) and serves as a powerful predictor of all-cause mortality and morbidity, reflecting the body’s capacity for oxygen utilization and consumption (Gonzales et al., 2021). Traditionally, assessing cardiopulmonary function and VO_2max_ typically requires the use of laboratory equipment, which is not only time-consuming and laborious but also complex in operation, limiting its application in daily life and practical exercise scenarios. However, with rapid technological advancements, the emergence of portable cardiopulmonary function measurement devices offers a new solution. The advent of these devices has made individual cardiopulmonary function assessment more convenient, flexible, and efficient. For instance, Elliot et al. measured the accuracy and reliability of heart rate and respiratory rate in elite cyclists wearing the Hexoskin wearable biometric vest during incremental load testing up to exhaustion (Elliot et al., 2019). Liu et al. validated the reliability and effectiveness of data such as heart rate, respiratory rate, multi-point skin temperature, and core temperature collected by subjects wearing the wearable device Equivital Life Monitor EQ02 during rest, low-intensity, and moderate-intensity physical activities (Liu et al., 2013). Furthermore, Villar et al. tested the effectiveness of the Hexoskin wearable vest compared to laboratory-standard equipment in measuring heart rate, respiratory rate, tidal volume, minute ventilation (VE), and hip joint movement intensity during lying, sitting, standing, and walking (Villar et al., 2015). However, the aforementioned devices also have certain limitations. Whether it is the Hexoskin or the Equivital Life Monitor EQ02 wearable devices, their method of measuring biometric data involves direct contact with the skin to collect physiological data, rather than directly capturing respiratory parameters. This leads to lower validity and reliability, particularly in the measurement of respiratory-related data, and can result in significant errors. For instance, both the magnitude of physical movement and body fat percentage can affect the accuracy of the Hexoskin device. During high-intensity exercise, the increased upper body movement may cause poor contact between the vest sensors and the skin, generating noise signals that affect measurement accuracy. For individuals with higher body fat, the contact between Hexoskin sensors and the skin might be better, thereby improving the reliability and validity of the measurements. In the case of the Equivital Life Monitor EQ02, each participant is equipped with six dermal patches, each costing $77, and a Jonah pill, also priced at $77. However, these are single-use items, which can become expensive and may have their data accuracy affected by sweat on the skin surface, especially in studies requiring multiple participants and multiple sensors. In contrast, the wearable device developed by our research group directly collects respiratory gas data from the body, offering higher measurement accuracy. Additionally, the device does not require disposable consumables, making it suitable for large-scale, repeated measurements across various populations. This makes it more widely applicable in fields such as healthcare, clinical settings, and scientific research.

In this context, the development and validation of portable cardiopulmonary function measurement devices hold great potential. Therefore, our research team has developed a portable cardiopulmonary function measurement device named “Booster,” which utilizes sequential respiration method and incorporates multiple sensors to monitor subjects’ minute ventilation, respiratory rate, tidal volume, real-time carbon dioxide concentration, and oxygen consumption during exercise. This product, with proprietary intellectual property rights, features small size, light weight, and absence of hose connections, making it suitable for monitoring exercise intensity, evaluating cardiopulmonary function, and providing exercise guidance. Additionally, the device comes with a dedicated cloud-based data management platform for managing user information, accessing exercise record data for each session, and guiding users in physical training. Users can view respiratory metabolism data at each time point of their exercise and query all previously measured exercise metabolism data. We have also developed a matching software called “Booster,” available for download on Android devices, which can be connected to smartphones or tablets via Bluetooth. The app displays the data from Booster tests, stores measurement results, and provides real-time display of measurement values on terminal devices. Users can simply open the app, enter their account, and log in to access the features.

The Cortex Metalyzer 3B (CORTEX, Leipzig, Germany) gas metabolism analyzer, equipped with built-in bidirectional turbine flow sensors, can monitor subjects’ minute ventilation for each breath in real-time. This method calculates changes in oxygen uptake by analyzing the actual gases exhaled and inhaled by the body during exercise, with high accuracy, making it the current mainstream method for measuring oxygen uptake (Adeel et al., 2022; Devereux et al., 2022; Lichti et al., 2023; Miranda et al., 2018). Compared to the Cortex Metalyzer 3B, “Booster” not only offers greater portability but also features easier operation and broader applicability across different populations and application scenarios. Additionally, “Booster” can significantly reduce measurement costs and improve measurement efficiency compared to the Cortex Metalyzer 3B system. In this study, we hypothesize that the newly developed portable cardiopulmonary measurement device, Booster, can provide measurement results consistent with the existing gold standard Cortex Metalyzer 3B system across various exercise intensities and modes, while also demonstrating good repeatability. However, we anticipate that Booster may exhibit some measurement errors during rest and extreme fatigue stages. Therefore, this study will delve into the design and functionality of the Booster portable cardiopulmonary function measurement device and compare its performance with the established fixed system, the Cortex Metalyzer 3B cardiopulmonary function testing system, to evaluate the validity and reliability of the Booster portable cardiopulmonary function measurement device. It is hoped that this will establish a foundation for its widespread application in exercise physiology research, clinical practice, and exercise performance monitoring.

## 2 Materials and methods

### 2.1 Study participants

**FIGURE 1:**
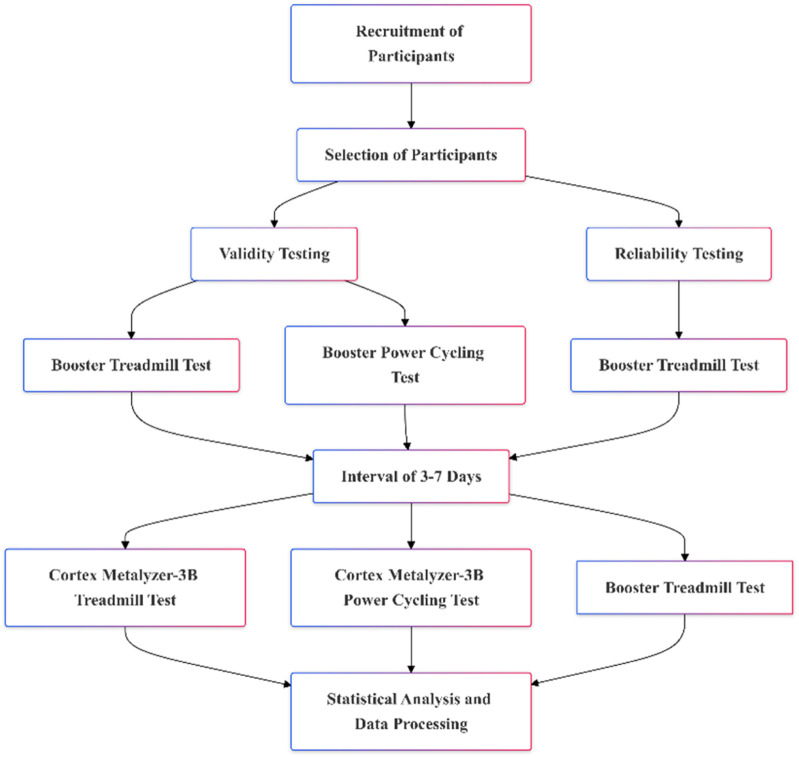
Study visit timeframe overview.

This study recruited 64 participants, all of whom underwent validity testing. Following this, 22 participants were randomly selected for reliability testing using a random number table. The tests were conducted at the Oxygen Metabolism and Risk Assessment Laboratory of Beijing Sport University. The laboratory environment was strictly controlled, with temperatures maintained at 24°C–27°C and humidity levels between 40% and 50%. Testing sessions were scheduled between 8:00 a.m. and 6:00 p.m. All participants were required to follow the same preparation standards before testing, including dietary, sleep, and exercise control. Additionally, each participant completed both tests within the same time frame (±1 h) to minimize the impact of time variations. The inclusion and exclusion criteria for participants were as follows: The inclusion criteria are: (1) Healthy adults aged between 18 and 35 years. (2) Physically healthy, with no cardiovascular or respiratory diseases or musculoskeletal disorders. (3) Regular exercise habits (exercising at least three times a week, each session lasting more than one hour). The exclusion criteria are: (1) Underwent surgery or treatment for cardiovascular or musculoskeletal systems. (2) Smokers, individuals currently using or long-term using medications that may affect cardiovascular or pulmonary function. (3) Regular consumption of coffee, tea, or alcoholic beverages. All participants were instructed to avoid intense exercise 24 h prior to testing and refrain from consuming coffee, tea, or alcoholic beverages 2 hours before the session. Participants were also advised to maintain their normal sleep patterns. Before the experiment commenced, they were briefed on the testing process and any related precautions. All participants provided written informed consent after being fully informed about the study details. In this study, we used the G*Power 3.1 software, a widely utilized statistical tool, to calculate the required sample size to achieve sufficient statistical power (Kang, 2021). Based on previous studies (Elliot et al., 2019; Goulet-Pelletier and Cousineau, 2018), the parameters considered include an effect size of 0.5, an alpha error probability (α) of 0.05, and a power (1-β error probability) of 0.95. The G*Power calculation indicated that the total sample size should be 54. The specific experimental procedure is shown in Figure 1.

### 2.2 Equipment description

The Booster portable cardiopulmonary function meter collects data using a breath-by-breath method. The device weighs approximately 300g and has dimensions of 180 mm × 100 mm × 100 mm. Its main body is made from PC + ABS material, and the silicone mask is medical-grade silicone. The front of the Booster features various sensors for detecting respiratory metabolism, including an oxygen sensor, a carbon dioxide sensor, a Bluetooth module (BLE Bluetooth 5.0), and a lithium polymer battery (500 mAh capacity). The sensors are based on MEMS technology, with the oxygen sensor being an electrochemical sensor and the carbon dioxide sensor being an infrared CO_2_ sensor. The device benefits from a small size, high precision, good stability, low power consumption, fast response time, and relatively low manufacturing cost. It operates continuously for ≥6 h, and the Bluetooth connection can reach up to 10 m without obstructions. The maximum measurement ranges are 0–80 mL/kg/min for oxygen consumption and 0–200 LPM for ventilation. The back part consists of a comfortable respiratory mask and head strap, which provides excellent fixation, securing the mask firmly to the face to prevent air leakage. For this study, we developed a specialized cloud-based data management platform to manage user information, access exercise records, and provide guidance and feedback on physical exercise, including exercise intensity and duration, for personalized exercise recommendations. We also developed a dedicated software application called “booster,” which can be downloaded on Android devices and installed on smartphones or tablets. This app displays and stores data from the Booster tests, with real-time measurements shown on the device. Users can access the app by opening it, entering their account details, and logging in. The Booster connects to the app via Bluetooth. Once the Booster portable cardiopulmonary function meter is worn, exercise data, including respiratory metabolism indicators such as VO_2_, VE, and CO_2_ concentration, as well as information on speed and distance, are displayed in real-time on the mobile app interface. During exercise, the app provides voice prompts at regular intervals, informing users of their speed, distance, and exercise intensity. We used the moving average method for data processing to calculate VO_2_ and VE. Additionally, before exercising, users can easily access the calibration function through the “calibration” button in the “booster” app. Users simply need to tap this button to start the calibration process. The “calibration” function relies on the internal calibration logic circuits and MCU processor of the device, featuring built-in chip-level calibration and compensation algorithms, factory calibration, and environmental calibration during use. Calibration is achieved through a combination of calibration gases, instrument calibration, and zero-point calibration. This ensures real-time and effective acquisition of sensor signals, accurate measurements, and strong interference resistance.

### 2.3 Testing protocols

#### 2.3.1 Incremental treadmill exercise protocol

The Bruce treadmill exercise protocol was employed for testing. After participants were equipped with the devices, they rested quietly for 3 min before testing to record data at rest. Subsequently, the exercise load was increased every 3 min until exhaustion, as outlined in Table 1. Heart rate and subjective fatigue level were recorded in the last 30 s of each stage (Yun et al., 2024; Garcin et al., 1998). Test personnel encouraged participants actively throughout the test to ensure accuracy and reminded them to relax and maintain a stable exercise rhythm. Upon reaching exhaustion, participants’ heart rate, subjective fatigue level, and exercise duration were recorded. Specially trained researchers were responsible for observing participants’ status to ensure their safety.

**TABLE 1:** Bruce treadmill load protocol.

Test stage	Speed (km/h)	Incline (°)	Duration (min)
Rest	0	0	3 min
Stage 1	2.7	10	3 min
Stage 2	4.0	12	3 min
Stage 3	5.4	14	3 min
Stage 4	6.7	16	3 min
Stage 5	8.0	18	3 min
Stage 6	8.8	20	3 min
Stage 7	9.6	22	3 min

The specific stopping criteria are as follows: Stopping criteria for exercise termination: (1) Rating of Perceived Exertion (RPE) ≥ 18; (2) Respiratory Exchange Ratio (RER) ≥ 1.10; (3) Achieving ≥ 90% of maximum heart rate (Max HR, calculated as 220 - age); (4) Plateauing of oxygen uptake, where oxygen uptake variation is less than 2 ml/min/kg for 2 mins of exercise, meeting three of these criteria indicates reaching maximal oxygen uptake. If participants exhibit symptoms such as difficulty breathing, grimacing, body swaying, or pale complexion, the experiment should be stopped immediately to ensure participant safety.

#### 2.3.2 Incremental intensity cycling ergometer exercise protocol

Participants began with a 3-min calm phase on the ergometer, initiating at 60W and escalating by 20W each minute until reaching exhaustion. During the test, participants were reminded to maintain a pedal cadence of 60 revolutions per minute. Heart rate and RPE values were recorded at the end of each stage, and immediate RPE and heart rate post-exercise were recorded, along with exercise duration. After exercise, data collected from both instruments were analyzed and processed uniformly using respective software (Ertl et al., 2016). Criteria for cessation mirrored those of the treadmill protocol, with the addition that failure to maintain cadence under encouragement was also a determinant for maximal oxygen uptake achievement.

### 2.4 Reliability and validity testing procedures and methods

#### 2.4.1 Reliability testing procedure and methods

Twenty-two participants participated in reliability testing. After wearing the Booster portable cardiopulmonary function measurement device, all participants completed one incremental treadmill test. After a 3–7-day interval for complete recovery, the same experimental procedure was repeated to test the reliability and consistency of the Booster portable cardiopulmonary function measurement device.

Data collected from the incremental treadmill tests with the Booster portable cardiopulmonary function measurement device were used for reliability analysis. Data from the last 2:30 to 3:00 min of each stage were analyzed for reliability. The coefficient of variation (CV) for both VE and VO_2_ in each exercise stage was calculated to assess reliability. The CV was calculated using the formula: CV = (standard deviation SD/mean) × 100% (McLaughlin et al., 1998). Specifically, based on previous studies (Liu et al., 2013; Villar et al., 2015; Hodges et al., 2005; Metsios et al., 2006), we consider a CV value of less than 10% to be ideal, indicating high stability and consistency in measurements. Conversely, a CV value equal to or greater than 10% is considered suboptimal, suggesting greater variability and lower reliability in the results.

Reliability was assessed based on data from two identical test procedures using the Booster system. The intraclass correlation coefficient (ICC) was calculated using a two-way random-effects (consistency) analysis of variance (ANOVA) model to evaluate the reliability of the Booster system, with ICC values interpreted as follows: ICC = 0.50–0.75 indicates moderate reliability, ICC = 0.75–0.90 indicates good reliability, and ICC >0.90 indicates excellent reliability (Koo and Li, 2016; Trumpf et al., 2020). A paired sample *t*-test was used to verify differences between the two tests. Bland-Altman plots were constructed to determine the 95% limits of agreement within the Booster system, providing a visual assessment of systematic bias between repeated tests (Stuckenschneider et al., 2020).

#### 2.4.2 Validity testing procedure and methods

Participants were divided into two groups: a treadmill exercise group (n = 46) and a cycling ergometer groups (n = 18), as shown in Table 2. Participants from both groups visited the laboratory twice, wearing either the Cortex Metalyzer-3B (CORTEX, Leipzig, Germany) or the Booster portable cardiopulmonary function measurement device to complete two exercise tests, with a 3–7-day interval between tests. The timing of the two tests was kept as similar as possible (±1 h).

**TABLE 2:** Grouping of participants.

Group	Age (years)	Height (cm)	Weight (kg)	BMI (kg/m^2^)
Treadmill Exercise Group (n = 46)	21.59 ± 3.44	175.00 ± 7.65	69.71 ± 11.4	22.66 ± 3.44
Cycling ergometer groups (n = 18)	19.94 ± 1.987	177.17 ± 5.36	72.56 ± 6.41	23.14 ± 2.55

The arithmetic mean of respiratory data collected during the last 30 s of each exercise stage (treadmill) or the last 20 s (Cycling ergometer) in the same exercise protocol was calculated to evaluate the validity of the Booster system. A paired sample *t*-test was used to test for mean differences in each measurement variable. The Pearson correlation coefficient (r) was used to evaluate the correlation between the same variables in different exercise stages between devices (r = 0.40–0.60 indicates moderate correlation, r = 0.60–0.79 indicates high correlation, and r ≥ 0.80 indicates high correlation) (Akoglu, 2018). The mean absolute difference (MAD = |measurement value-standard value|) and mean absolute percentage error (MAPE=(|measurement value-standard value|)/standard value*100%) were calculated to analyze the error between the Booster and Cortex devices. Using linear regression to analyze and assess the consistency of VE and VO_2_ between the Cortex Metalyzer-3B and Booster systems. Constructing Bland-Altman plots to determine the 95% limits of agreement between the Cortex Metalyzer-3B and Booster systems, as well as conducting intra-class correlation coefficient (ICC) consistency analysis.

### 2.5 Data processing

Statistical analysis of experimental data was conducted using SPSS 24.0 statistical analysis software. After normal distribution and homogeneity of variance tests, all data results were presented as mean ± standard deviation (X±SD). A significance level of *p* < 0.05 indicated statistical significance, while *p* < 0.01 indicated highly significant differences. All confidence intervals were set at 95%.

## 3 Results

### 3.1 Basic characteristics of participants

The basic characteristics of the participants in this study are shown in Table 3. The participants were divided into two groups: the validity testing group (n = 64) and the reliability testing group (n = 22). There were no significant differences between the two groups in terms of baseline levels of Age, Height, Weight, BMI, VO_2max_, and VE_max_.

**TABLE 3:** Basic information of participants.

Group	Age (years)	Height (cm)	Weight (kg)	BMI (kg/m^2^)	VO_2max_ (mL/kg/min)	VE_max_ (L/min)
Validity testing group (n = 64)	21.13 ± 3.17	175.67 ± 7.1	70.5 ± 10.28	22.8 ± 2.71	47.86 ± 7.82	110.07 ± 27.61
Reliability testing group (n = 22)	21.09 ± 3.17	175.91 ± 5.46	68.8 ± 9.08	22.21 ± 2.55	50.24 ± 6.49	109.39 ± 21.33

### 3.2 Reliability analysis

#### 3.2.1 Reliability analysis of the Booster portable cardiopulmonary function meter

The results of VO_2_ and minute ventilation (VE) measured by the Booster at different exercise stages are shown in Tables 4, 5. The paired sample *t*-test results show no significant differences in VO_2_ and VE between the two measurements with the Booster in various stages of the treadmill test (*p* > 0.05).

Statistical analysis of the coefficient of variation (CV%) of VO_2_ and VE measured by the Booster at different exercise intensities revealed that the CV% of VO_2_ ranged from 2.10 ± 1.22 to 2.22 ± 1.39, while the CV% of VE ranged from 13.23 ± 6.97 (at rest) to 2.2 ± 1.39 to 2.79 ± 2.28.

**TABLE 4:** Reliability analysis of VO_2_ measurement by the Booster portable cardiopulmonary function meter.

Speed	Rest	2.7 km/h	4 km/h	5.4 km/h	6.7 km/h	Total
Booster1	6.43 ± 2.15	17.62 ± 1.92	24.19 ± 2.00	34.3 ± 2.74	48.7 ± 4.40	
Booster2	6.49 ± 1.99	18.47 ± 2.93	24.71 ± 3.56	36.4 ± 5.25	49.31 ± 6.36	
CV(%)	5.14 ± 2.56	2.91 ± 1.81	2.10 ± 1.22	2.79 ± 2.00	2.61 ± 2.78	
ICC(p)	0.031 (*p* = 0.89)	0.185 (*p* = 0.410)	0.509 (*p* = 0.015)	0.814 (*p* < 0.001)	0.762 (*p* < 0.001)	0.797
*p* value	0.917	0.228	0.439	0.426	0.376	

Note: Booster1 represents the VO_2_ data measured by the Booster Portable Cardiopulmonary Function Tester in the first measurement; Booster2 represents the VO_2_ data measured by the Booster Portable Cardiopulmonary Function Tester in the second measurement; CV, coefficient of variation; ICC, intraclass correlation coefficient.

**TABLE 5:** Reliability analysis of VE measurement by the Booster portable cardiopulmonary function meter.

Speed	Rest	2.7 km/h	4 km/h	5.4 km/h	6.7 km/h	Total
Booster1	11.27 ± 3.46	25.22 ± 4.85	40.24 ± 6.53	66.72 ± 11.81	107.14 ± 20.25	
Booster2	10.94 ± 2.75	24.92 ± 4.02	39.48 ± 6.95	67.06 ± 13.79	105.26 ± 22.57	
CV(%)	13.23 ± 6.97	2.71 ± 1.12	2.22 ± 1.39	2.79 ± 2.28	2.61 ± 2.78	
ICC(p)	0.767 (*p* < 0.001)	0.807 (*p* < 0.001)	0.776 (*p* < 0.001)	0.859 (*p* < 0.001)	0.937 (*p* < 0.001)	0.832
*p* value	0.598	0.626	0.439	0.816	0.794	

Note: Booster1 represents the VE, data measured by the Booster Portable Cardiopulmonary Function Tester in the first measurement; Booster2 represents the VE, data measured by the Booster Portable Cardiopulmonary Function Tester in the second measurement; CV, coefficient of variation; ICC, intraclass correlation coefficient.

After ICC analysis of the repeated measurements, the overall ICC for VO_2_ during the entire exercise process was 0.797, and for VE, it was 0.832, both greater than 0.75. However, during rest, the ICC for VO_2_ was 0.031, and for VE, the CV% was 13.23 ± 6.97, showing a significant difference from the overall values. Additionally, at different exercise intensities, the consistency of Booster measurements for VO_2_ and VE mostly reached a good level (ICC >0.75, *p* < 0.001).

**FIGURE 2:**
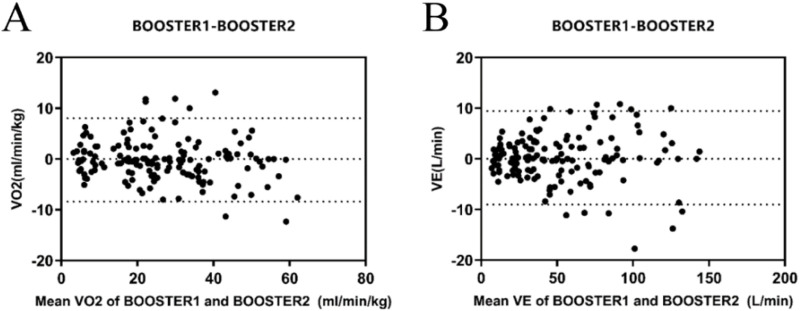
Reliability Analysis of the Booster Portable Cardiopulmonary Function Tester **(A)** Bland-Altman plot of VO_2_ measurements between the two consecutive measurements with the Booster Portable Cardiopulmonary Function Tester; **(B)** Bland-Altman plot of VE measurements between the two consecutive measurements with the Booster Portable Cardiopulmonary Function Tester.

The Bland-Altman plots analysis of the two exercise tests showed that for VO_2_ (Figure 2A) and VE (Figure 2B) measurements, the average absolute errors for the Booster were −0.165 mL/kg/min and 0.22 L/min, respectively, with 95% confidence intervals ranging from −8.3 to 8.0 mL/kg/min and 9.00–9.43 L/min.

#### 3.2.2 Treadmill exercise test: reliability analysis of VE_max_ and VO_2max_

The results of VO_2max_ and VE_max_ achieved during exhaustion in treadmill tests with subjects wearing the Booster device on two occasions are presented below (Table 6). Pearson correlation analysis demonstrates a correlation coefficient of 0.762 for VO_2max_ and 0.800 for VE_max_ between the two tests, indicating a high correlation between the measured VO_2max_ and VE_max_ by the Booster device (r > 0.75). Furthermore, the ICC intra-class correlation analysis reveals coefficients greater than 0.75 for both VO_2max_ and VE_max_ in the two measurements.

**TABLE 6:** Analysis of VE_max_ and VO_2max_ in treadmill exercise test.

Instrument	VO_2max_ (mL/kg/min)	VE_max_ (L/min)
Booster1	49.65 ± 5.89	111.34 ± 19.93
Booster2	50.83 ± 7.09	107.43 ± 22.72
Pearson’s *r*	0.762	0.800
ICC(*P*)	0.753(*p* < 0.0001)	0.793(*p* < 0.0001)

Note: Booster1 represents the VO_2_ data measured by the Booster Portable Cardiopulmonary Function Tester in the first measurement; Booster2 represents the VO_2_ data measured by the Booster Portable Cardiopulmonary Function Tester in the second measurement; Pearson’s r: correlation coefficient; ICC, intra-class correlation coefficient; VO_2max_, maximal oxygen consumption; VE_max_, maximal ventilation.

### 3.3 Validity Analysis

#### 3.3.1 Validity Analysis of incremental intensity treadmill exercise

In the incremental intensity treadmill tests, the Booster exhibited very strong correlations with the Cortex system for VO_2_ (Table 7) and VE (Table 8) measurements, with Pearson correlation coefficients of 0.991 and 0.976, respectively (r > 0.75). Paired sample t-tests showed no significant differences in VO_2_ and VE values measured by Booster and Cortex Metalyzer 3B throughout the treadmill test process (*p* > 0.05). However, significant differences were found in measurements at rest (0 km/h) for VO_2_ and VE between the two systems (*p* < 0.01). The average MAPE (%) for VO_2_ was 26.37% ± 24.95, and for VE it was 17.05% ± 11.88, suggesting notable discrepancies between the two systems.

**TABLE 7:** Validity analysis of VO_2_ measurements during treadmill exercise.

Speed	0 km/h	2.7 km/h	4 km/h	5.4 km/h	6.7 km/h	Total
Cortex	5.30 ± 1.14	17.04 ± 1.63	23.98 ± 1.99	34.99 ± 2.97	47.35 ± 4.87	
Booster	6.06 ± 1.99	17.42 ± 1.95	24.18 ± 2.21	35.03 ± 3.84	48.42 ± 5.23	
Pearson’s *r*	0.388	0.511	0.634	0.490	0.751	0.991
*P*	0.001	0.101	0.596	0.097	0.531	0.054
MAD	1.35 ± 1.14	1.48 ± 1.05	1.45 ± 1.07	2.77 ± 2.13	2.57 ± 2.25	
MAPE (%)	26.37 ± 24.95	8.89 ± 6.57	5.99 ± 4.21	7.91 ± 6.24	6.41 ± 4.42	

Note:Pearson’s r: correlation coefficient; MAD, mean absolute deviation; MAPE (%), mean absolute percentage error.

**TABLE 8:** Validity analysis of VE (L/min) measurements during treadmill exercise.

Speed	0 km/h	2.7 km/h	4 km/h	5.4 km/h	6.7 km/h	Total
Cortex	12.59 ± 2.66	27.60 ± 4.75	41.35 ± 6.72	68.10 ± 12.15	107.87 ± 21.13	
Booster	11.32 ± 3.24	26.69 ± 5.56	41.74 ± 8.27	69.45 ± 14.24	108.32 ± 21.00	
Pearson’s *r*	0.676	0.751	0.801	0.928	0.978	0.976
*P*	0.008	0.151	0.461	0.95	0.068	0.147
MAD	2.18 ± 1.62	2.90 ± 2.46	3.79 ± 3.16	4.25 ± 3.56	2.87 ± 2.82	
MAPE (%)	17.05 ± 11.88	10.55 ± 8.34	8.86 ± 6.99	6.00 ± 4.56	3.23 ± 2.76	

Note:Pearson’s r: correlation coefficient; MAD, mean absolute deviation; MAPE (%), mean absolute percentage error.

**FIGURE 3:**
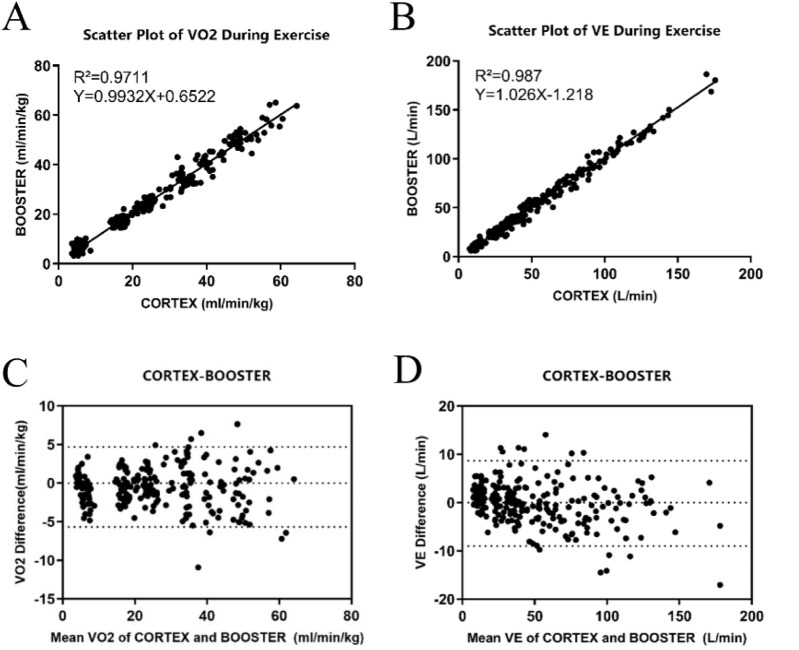
Validity Analysis of the Booster Portable Cardiopulmonary Function Meter **(A)** Linear regression curve of VO_2_ validity during treadmill exercise between Booster and Cortex; **(B)** Linear regression curve of VE validity during treadmill exercise between Booster and Cortex; **(C)** Bland-Altman analysis plot of VO_2_ validity during treadmill exercise between Booster and Cortex; **(D)** Bland-Altman analysis plot of VE validity during treadmill exercise between Booster and Cortex.

The linear regression analysis of both datasets (Figure 3A, B) indicated high correlation between Booster and Cortex Metalyzer 3B during the incremental intensity treadmill exercise for both VO_2_ and VE. The correlation coefficient R^2^ for VO_2_ was 0.971 with the regression equation Y = 0.993X+0.652, which was statistically significant (*p* < 0.01). Similarly, VE had a correlation coefficient R^2^ of 0.987 with the regression equation Y = 1.026X-1.218, also significant (*p* < 0.01).

Figures 3C, D display the Bland-Altman analysis for VO_2_ and VE measurements during treadmill exercise, respectively. In Figure 3C, most VO_2_ measurement points were within the 95% limits of agreement, indicating that the treadmill VO_2_ measurements between the two systems are within an acceptable range, with most points lying within 1.96 standard deviations of the mean. Figure 3D shows that the mean difference for VE measurements was −0.15 L/min, with a standard deviation of 4.51 L/min and limits of agreement from −9.02 L/min to 8.69 L/min.

#### 3.3.2 Validity Analysis of incremental intensity cycling ergometer exercise

For the entire exercise process of incremental intensity cycling ergometer tests, the overall Pearson correlation coefficients for VO_2_ and VE were greater than 0.94, at 0.944 and 0.984, respectively, indicating a high correlation between the two devices. Compared to the Cortex Metalyzer 3B, there were no significant differences in the overall VO_2_ measurements by Booster (*p* > 0.05). However, at 80W, significant differences were observed in VO_2_ measurements (*p* < 0.01). Significant differences were also noted in VE measurements, particularly at low exercise intensities from rest to 100W (*p* < 0.05), as detailed in Tables 9, 10. When comparing the MAPE (%) between the two devices, it was noted that the measurement discrepancies for VO_2_ from rest up to 120W were considerable, exceeding 10%. For VE measurements, only the values at rest and 60W were greater than 10%.

**TABLE 9:** Validity Comparison of VO_2_ Measurement in Cycling ergometer Exercise.

Speed	0W	60W	80W	100W	120W	140W	160W	180W	200W	Total
Cortex	5.36 ± 0.88	13.26 ± 2.08	18.04 ± 1.75	21.39 ± 1.90	24.42 ± 2.47	27.89 ± 3.07	31.25 ± 3.07	35.73 ± 3.38	38.46 ± 3.02	
Booster	5.45 ± 1.79	11.99 ± 3.55	17.49 ± 3.25	21.20 ± 3.60	23.50 ± 3.82	27.27 ± 3.48	31.24 ± 4.47	35.87 ± 5.37	38.66 ± 6.00	
Pearson’s r	0.367	0.603	0.569	0.651	0.798	0.725	0.824	0.917	0.934	0.944
P	0.825	0.092	0.001	0.786	0.281	0.428	0.988	0.909	0.896	0.204
MAD	1.26 ± 1.12	2.55 ± 1.98	2.04 ± 1.39	2.50 ± 1.32	3.07 ± 1.75	2.61 ± 1.94	3.24 ± 3.00	3.70 ± 3.55	4.09 ± 3.11	
MAPE (%)	23.73 ± 20.76	19.47 ± 15.03	11.39 ± 7.78	11.89 ± 6.61	12.9 ± 1.94	9.74 ± 7.87	10.22 ± 9.38	10.11 ± 9.38	10.74 ± 8.20	

Note: Pearson’s r: correlation coefficient; MAD, mean absolute deviation; MAPE (%), mean absolute percentage error.

**TABLE 10:** Comparative validity of VE measurements during cycling ergometer exercise between Booster and Cortex.

Speed	0W	60W	80W	100W	120W	140W	160W	180W	200W	Total
Cortex	13.22 ± 2.36	23.26 ± 2.60	32.03 ± 3.61	39.78 ± 4.39	47.89 ± 5.90	55.92 ± 7.74	66.54 ± 9.81	83.04 ± 14.65	95.63 ± 19.89	
Booster	11.49 ± 1.95	20.76 ± 3.76	30.01 ± 4.37	37.83 ± 4.83	46.37 ± 6.84	55.19 ± 8.06	64.94 ± 10.93	81.67 ± 16.07	93.30 ± 18.65	
Pearson’s *r*	0.336	0.532	0.377	0.604	0.451	0.513	0.338	0.364	0.481	0.984
P	0.008	0.003	0.036	0.048	0.137	0.603	0.293	0.374	0.259	0.001
MAD	2.39 ± 1.76	2.87 ± 2.65	3.20 ± 2.76	3.37 ± 2.66	3.38 ± 2.74	4.84 ± 3.19	5.12 ± 3.73	5.27 ± 3.68	5.42 ± 4.92	
MAPE (%)	17.30 ± 11.24	12.21 ± 11.28	9.98 ± 8.59	8.22 ± 6.25	6.87 ± 4.98	8.50 ± 3.73	7.62 ± 5.24	6.38 ± 4.75	5.35 ± 4.36	

Note: Pearson’s r: the correlation coefficient; MAD, mean absolute deviation; MAPE (%), mean absolute percentage error.

**TABLE 11:** HR, RPE, and test duration information in treadmill exercise.

Instrument	HR_rest_	HR_max_	RPE_rest_	RPE_max_	Time(s)
Booster	72.73 ± 12.36	190.54 ± 6.38	6.32 ± 0.74	16.30 ± 1.91	721.47 ± 33.54
Cortex	73.92 ± 11.56	188.93 ± 6.74	6.48 ± 0.76	15.72 ± 1.37	739.51 ± 32.79∗
Pearson’s *r*	0.783	0.825	0.400	0.608	0.873

Note: Pearson’s r: the correlation coefficient; HR_rest_, resting heart rate; HR_max_, maximum heart rate; RPE_rest_, resting RPE; RPE_max_, maximum RPE. *p < 0.05.

**TABLE 12:** VO_2max_ and VE_max_ during test process.

Instrument	VO_2max_ (treadmill)	VE_max_ (treadmill)	VO_2max_ (cycling)	VE_max_ (cycling)
Booster	49.32 ± 7.80	111.62 ± 28.46	41.76 ± 5.92	100.63 ± 17.02
Cortex	46.43 ± 7.81_∗∗_	108.51 ± 26.76_∗∗_	39.67 ± 4.46	101.07 ± 17.42_∗∗_
Pearson’s *r*	0.854	0.984	0.400	0.908
ICC	0.854	0.983	0.384	0.907
*P*	0.001	0.001	0.052	0.001

Note: Pearson’s r represents the correlation coefficient; ICC, indicates the intraclass correlation coefficient; *p < 0.05, **p < 0.01, ***p < 0.001; VO_2max_, maximal oxygen uptake; VE_max_:maximal ventilation volume.

**FIGURE 4:**
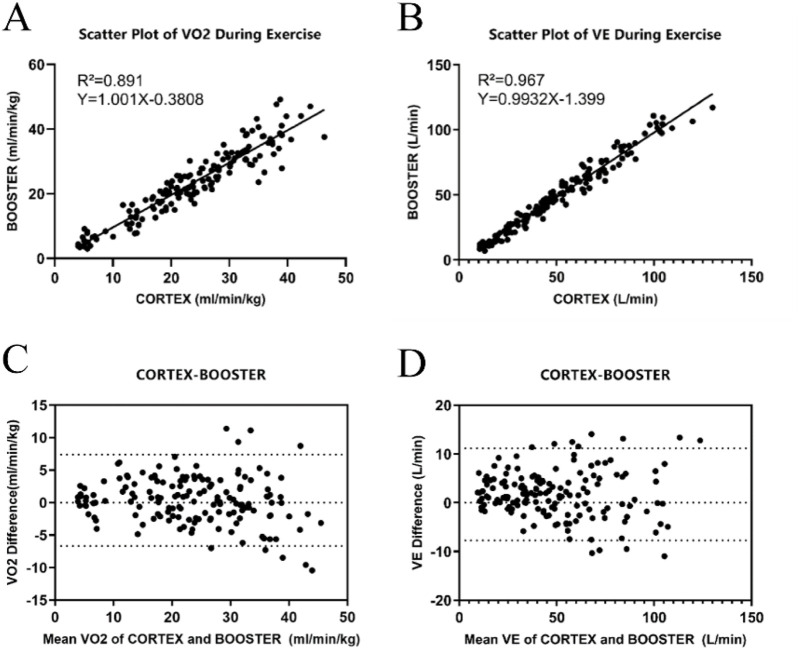
Validity Analysis of the Booster Portable Cardiopulmonary Function Meter **(A)** Linear regression curve for the validity of VO_2_ measurements during cycling ergometer exercise between Booster and Cortex. **(B)** Linear regression curve for the validity of VE measurements during cycling ergometer exercise between Booster and Cortex. **(C)** Bland-Altman plot for the validity of VO_2_ measurements during cycling ergometer exercise between Booster and Cortex. **(D)** Bland-Altman plot for the validity of VE measurements during cycling ergometer exercise between Booster and Cortex.

Ordinary linear regression analysis revealed that the Booster showed high correlation for VO_2_ and VE during the incremental intensity cycling ergometer tests. The scatterplot regression equation for VO_2_ was Y = 1.001X-0.3808 with a correlation coefficient R^2^ of 0.891, and for VE, the regression equation was Y = 0.993X-1.399 with a correlation coefficient R^2^ of 0.967, both showing statistical significance (*p* < 0.01). Figures 4A, B would display the linear regression analysis results for VO_2_ and VE measured by the two devices.

Figures 4C, D show the Bland-Altman analysis results of VO_2_ and VE measurements by the two devices during Cycling ergometer exercise. The mean difference in VO_2_ measurement between the two devices was 1.73 mL/(minkg), with a standard deviation of 4.82 mL/(minkg) and limits of agreement (−7.72 mL/(minkg), 11.19 mL/(minkg)). The mean difference in VE measurement was 0.36 L/min, with a standard deviation of 3.59 L/min and limits of agreement (−6.67 L/min, 7.40 L/min).

#### 3.3.3 Analysis of data at exhaustion

The analysis of heart rate (HR), perceived exertion (RPE), and exercise duration at exhaustion during treadmill exercise is presented in Table 11. There were no significant differences between the Booster and Cortex systems in measuring resting heart rate (HR_rest_), maximum heart rate (HR_max_), resting perceived exertion (RPE_rest_), or maximum perceived exertion (RPE_max_) (*p* > 0.05). However, there was a significant difference in the completion time between the two tests, with the Cortex measuring a significantly longer exhaustion time than the Booster (*p* < 0.05).

Table 12 shows significant differences between the two devices at the point of exhaustion for VE_max_ and VO_2max_ during treadmill exercise, as well as for VE_max_ during cycling ergometer exercise (*p* < 0.01), while there were no significant differences for VO_2max_ during cycling. Pearson correlation analysis showed low correlation for cycling VO_2max_ (r < 0.60) and high correlation for treadmill VE_max_, treadmill VO_2max_, and cycling VE_max_ (r > 0.75). ICC coefficients indicated poor consistency for cycling VO_2max_, while treadmill VO_2max_, treadmill VE_max_, and cycling VE_max_ all had ICC values greater than 0.75.

## 4 Discussion

This study compared the Booster portable cardiopulmonary function tester with the German Cortex Metalyzer-3B system to validate the reliability and validity of the Booster in measuring minute VE and VO_2_ during incremental intensity treadmill and cycling ergometer tests. Hodges et al.’s research (Hodges et al., 2005) indicated that when cardiopulmonary testing devices have a certain level of reliability and subjects undergo the same exercise protocol on the same device, the coefficient of variation (CV) of test parameters should be within an acceptable range of 9% or less. This study found that, except for the higher-than-acceptable CV value of VE during rest (13.23 ± 6.97), the CVs of VO_2_ and VE measured during other exercise stages were within an acceptable range of error. Similarly, the analysis based on ICC values also showed the reliability of the system, as all subjects underwent the same exercise protocol on the same device, further confirming the reliability of the Booster. Moreover, the analysis using Bland-Altman plots and linear regression intuitively validated its reliability.

Changes in the body’s metabolism throughout the day may also affect the reliability of repeated measurements. For example, Gasic et al.’s study (Gasic et al., 1997) demonstrated that when repeated resting measurements are performed, even with intervals shortened to 15–20 min, the coefficients of variation for VO_2_ and VCO_2_ are 3.7% and 4.6%, respectively. Larger differences may occur when the tests are repeated over several days. Thus, over time, the combination of biological and technical variations may lead to differences within subjects. A study (Perez-Suarez et al., 2018) examined the biological and technical variability of VO_2_, VCO_2_, and VE measured by a wearable device, COSMED K5, finding that the accuracy of COSMED K5 may vary with different measurement modes, especially in mixed breathing modes, with VO_2_ at rest being overestimated by approximately 13.4%. The Booster system also showed lower reliability when measuring VE and VO_2_ at rest and slower walking speeds, as reflected in higher CVs and lower ICC scores. This may be due to the inefficiency of slow walking or the calculation method of CVs. Rock et al. found (Rock et al., 2018) that the metabolic cost of walking follows a “J” shaped curve, with the lowest point occurring near slow walking speeds. The increased energy expenditure during slow walking may be due to larger changes in stride length. In the current study, the reliability of the Booster decreased at slower walking speeds, possibly due to increased variability from slower walking speeds, metabolic cost, and stride length. Additionally, according to the function of CV calculation, CVs may be lower at higher walking speeds. As the mean value increases, variability decreases or remains the same, resulting in a lower coefficient of variation.

When measuring the complete treadmill exercise process, we found a highly correlated relationship between the Booster and Cortex systems, with Pearson correlation coefficients exceeding 0.97, indicating a very high overall correlation. Further observation at different exercise intensity stages revealed that as the exercise intensity increased, the correlation between the two devices also increased. This suggests that the accuracy of the Booster’s measurements improves with increasing exercise intensity, a phenomenon similarly validated in reliability analysis. In this study, we explored the validity of VE and VO_2_ at different speed stages during treadmill exercise using the Booster. It is noteworthy that when subjects wore the Booster to exhaustion during incremental treadmill exercise, the ICC coefficients of VE_max_ and VO_2max_ measured by both devices were greater than 0.75, indicating high reliability, and there were no significant differences between the two tests. This indicates good reliability of the Booster in measuring VE and VO_2_ at exhaustion.

In daily training, heart rate is commonly used as an objective indicator of exercise intensity, while ratings of perceived exertion (RPE) are used as a subjective assessment of individual internal and external environmental information (Morishita et al., 2019; Rago et al., 2022; Morishita et al., 2018). In this study, there were no significant differences in resting RPE and heart rate values before testing, indicating consistent baseline conditions among subjects. The maximum heart rates measured by the Booster and Cortex devices were (190.54 ± 6.38) beats/min and (188.93 ± 6.74) beats/min, respectively, with no significant differences (*p* > 0.05), and they were significantly positively correlated (r = 0.825). The maximum RPE reached was 16.30 ± 1.91 for the Booster and 15.72 ± 1.37 for the Cortex, with no significant differences (*p* > 0.05). However, we noticed that in the same incremental load exercise mode, subjects wore the Booster for nearly 20 s less than the Cortex, which we speculate may be due to the design of the exhalation tube used during instrument testing and its impact on breathing patterns. Our design of the Booster involves mounting sensors on the front of the mask, which increases resistance during inhalation. While this may not be very noticeable during low to moderate intensity exercise, it could have a certain degree of impact during vigorous exercise, especially when respiratory frequency is high and flow rate is fast. This phenomenon provides valuable reference for the optimization and improvement of the Booster, helping us better identify and address potential issues.

In conclusion, through Pearson correlation coefficient analysis, paired sample *t*-test, linear regression analysis, and Bland-Altman plot analysis of VE and VO_2_ measured by the Booster portable cardiopulmonary function test device and the Cortex Metalyzer-3B cardiopulmonary function test system, we found that the two systems exhibit very high reliability and validity in measuring VE and VO_2_. Although there are some differences in VE and VO_2_ measurements compared to the Cortex, the Booster, as a new type of portable cardiopulmonary function tester, is compact, easy to operate, and under the same measurement accuracy, it can achieve lower measurement costs. It is suitable for more exercise scenarios and for conducting multiple tests and exercise training for large populations. Additionally, it is equipped with a mobile/tablet application and a cloud-based data processing platform. The Booster portable cardiopulmonary function tester can connect to a mobile phone or tablet via Bluetooth. During exercise, users can view real-time respiratory metabolism and exercise data on their mobile devices. After the exercise, the data can be transmitted to the cloud, where professional personnel can analyze and process the exercise data, providing users with exercise recommendations. It is expected that this device will have broader applications in healthcare, clinical settings, and scientific research.

## 5 Conclusion

Compared to the Cortex Metalyzer-3B, the Booster portable cardiopulmonary function measurement demonstrates high reliability and validity in measuring VE and VO_2_ during exercise processes. However, some errors may occur when measuring at rest or during high-intensity exercise.

## 6 Limitations

To improve the consistency of Booster’s performance across all exercise intensities, we plan to increase the sample size in future studies, including participants of different genders and ages, and to conduct tests across a broader range of exercise intensities. Additionally, in subsequent studies, we intend to include more parameters, such as CO_2_ concentration, tidal volume, and respiratory rate, in our data analysis. We will focus on optimizing the algorithm and hardware design to reduce measurement errors under extreme conditions and further enhance Booster’s repeatability. We are confident that these measures will strengthen Booster’s reliability, making it a more dependable tool for cardiopulmonary function assessment in various practical applications.

## Data Availability

The raw data supporting the conclusions of this article will be made available by the authors, without undue reservation.
