# Supplementary figures and images for: Study on the stability and accuracy of the new Booster portable cardiopulmonary function meter
Source: Front Physiol. 2025 Jan 8;15:1453942. doi: 10.3389/fphys.2024.1453942 (PMC11751217; doi:10.3389/fphys.2024.1453942)

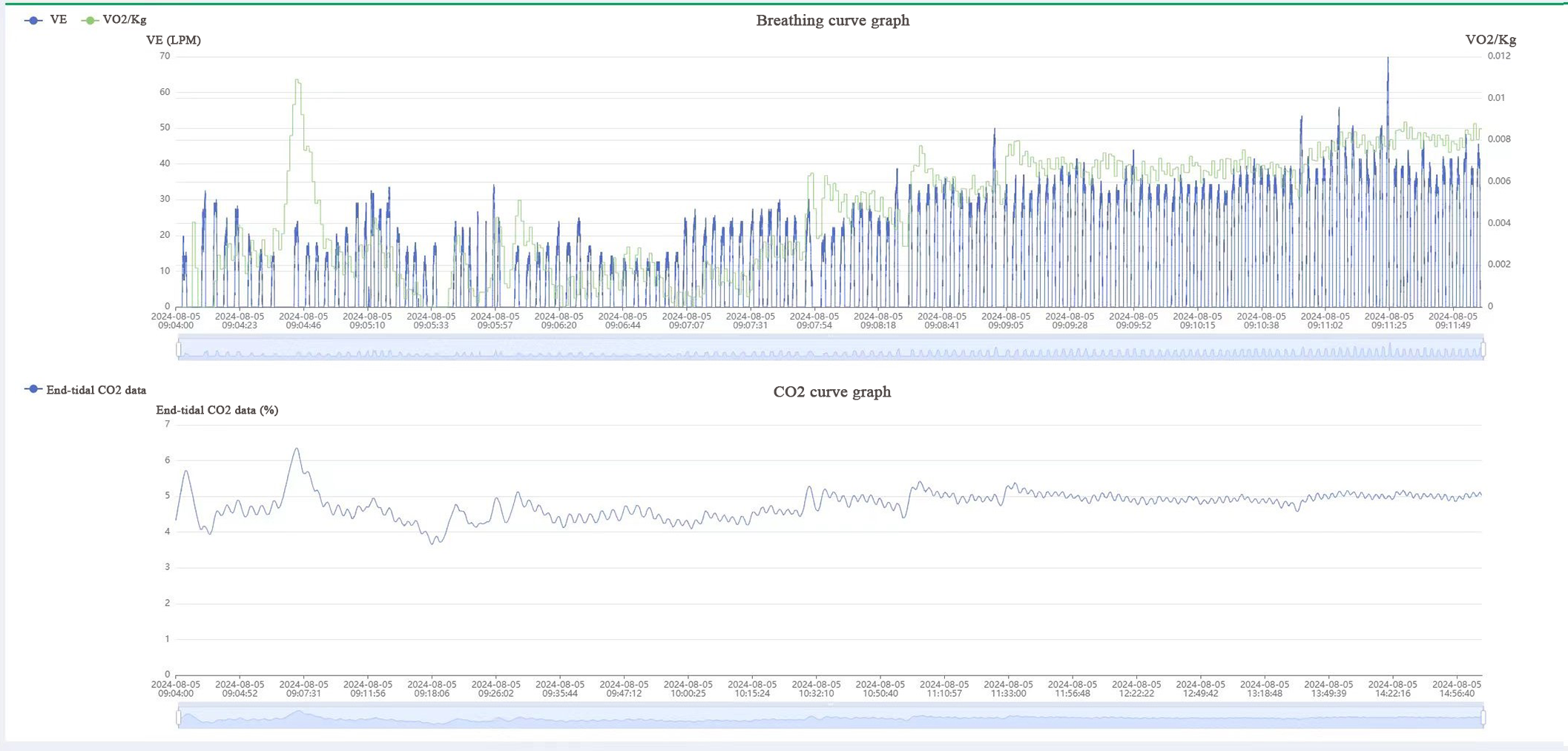

Supplement: Supplementary file 1 [file Image2.tif]

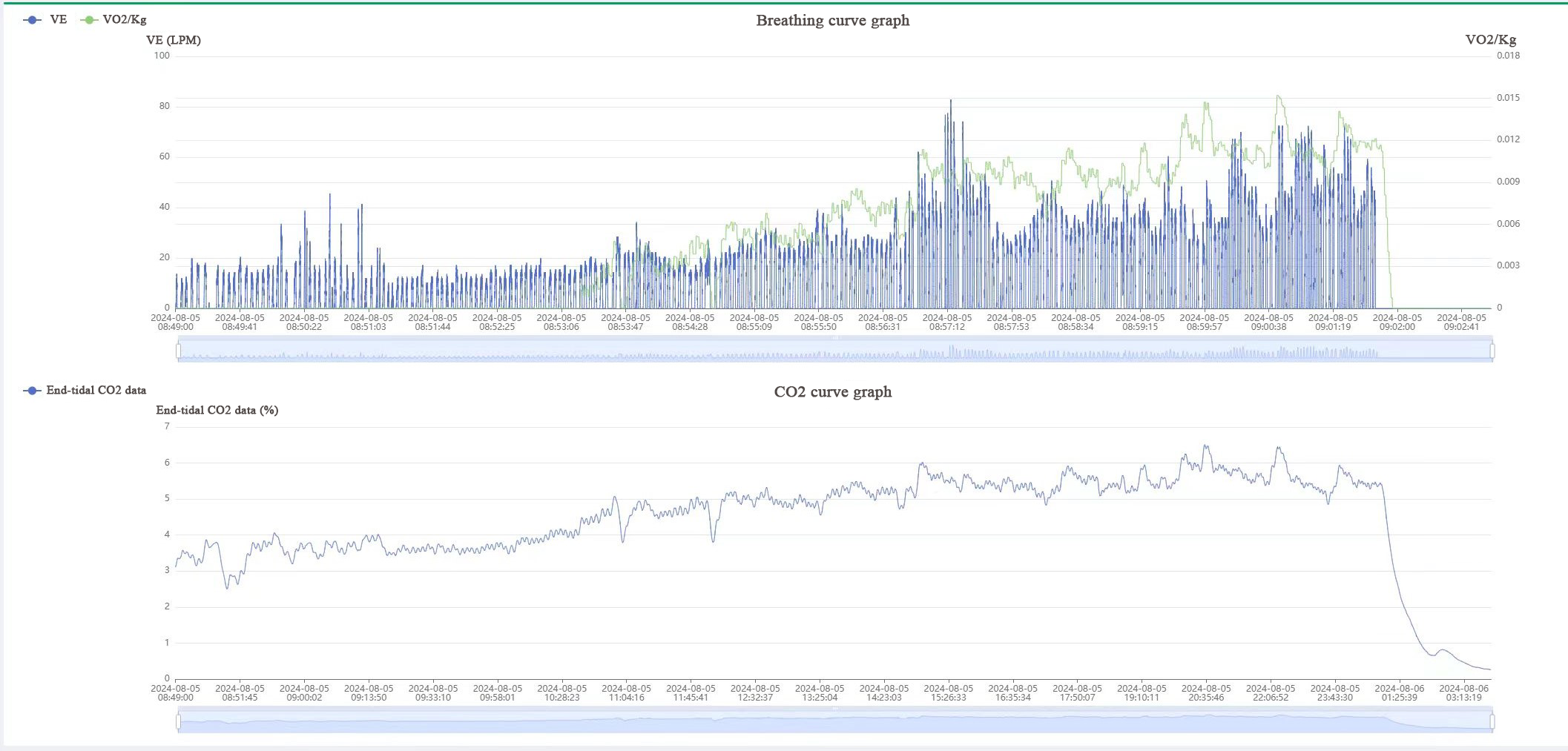

Supplement: Supplementary file 2 [file Image1.tif]
